# Decompressive craniectomy combined with temporal pole resection in the treatment of massive cerebral infarction

**DOI:** 10.1186/s12883-022-02688-0

**Published:** 2022-05-03

**Authors:** Wenchao Lu, Dong Jia, Yanchang Qin

**Affiliations:** Department of Neurosurgery, the Xi’an Daxing Hospital, No. 353 Laodong North Road, Xi’an, 710000 Shaanxi Province China

**Keywords:** Massive Cerebral Infarction, Decompressive Craniectomy, Intracranial Pressure, Stroke

## Abstract

**Objective:**

To evaluate the efficacy and prognosis of decompressive craniectomy combined with temporal pole resection in the treatment of massive cerebral infarction, in order to provide basis for treatment selection.

**Methods:**

The clinical data of the patient with massive cerebral infarction treated in our hospital from January 2015 to December 2018 were analyzed retrospectively. According to the surgical methods, the patients were divided into control group (decompressive craniectomy) and study group (decompressive craniectomy + temporal pole resection). Intracranial pressure monitoring devices were placed in both groups. The NIHSS scores of the two groups before and 14 days after operation, the changes of intracranial pressure, length of hospital stay, length of NICU, mortality and modified Rankin scale before and after treatment were compared between the two groups.

**Results:**

The NIHSS score of the two groups after operation was lower than that before operation, and the NIHSS score of the study group was significantly lower than that of the control group (*P* < 0.05); The intracranial pressure in the study group was significantly lower than that in the control group (*P* < 0.05); One month after operation, the mortality of the study group (13.0%) was lower than that of the control group (27.8%). After one year of follow-up, the mortality of the study group (21.7%) was significantly lower than that of the control group (38.8%) (*P* < 0.05); The scores of mRS in the two groups were significantly improved compared with those before treatment (*P* < 0.05), and the scores of mRS in the study group were better than those in the control group (*P* < 0.05).

**Conclusion:**

Decompressive craniectomy combined with temporal pole resection has a better effect in the treatment of patients with massive cerebral infarction. It has good decompression effect, the postoperative intracranial pressure is well controlled, and significantly reduced the mortality. So it has better clinical application value.

## Introduce

Massive cerebral infarction (MCI) is an infarction in the blood supply area of the middle cerebral artery≥2/3, with or without infarction in the blood supply area of the anterior cerebral artery / posterior cerebral artery. The incidence rate accounts for about 10% of all ischemic stroke. MCI has the characteristics of serious condition, rapid progress of disease course, difficult treatment and poor prognosis [1]. Conservative treatment is mainly to prevent recurrent infarction, reduce brain edema, decrease intracranial pressure and promote the recovery of neurological function, but its curative effect is slow and poor. With the progress of MCI, brain edema will continue to worsen, brain tissue will be compressed and displaced, cerebral hernia formation, and life-threatening. At the same time, malignant high intracranial pressure is formed, the cerebral perfusion pressure decreases, which further aggravates cerebral ischemia and necrosis, thus forming a vicious circle [2]. Clinical studies have shown that decompressive craniectomy (DC) can break this vicious circle, reduce intracranial pressure and relieve brain stem compression, significantly improve the prognosis of patients, and effectively reduce the disability rate and mortality [3]. However, the traditional DC is remove part of the skull of frontal, temporal and parietal on the affected side, cut the dura and suture it with tension reduction, so as to release the swollen brain tissue bulge outward and reduce intracranial pressure. However, some patients also died after DC, because of the continuous aggravation of brain edema. At present, there is no unified international standard on whether it is necessary to remove the temporal pole for internal decompression [4–6]. This study analyzed the efficacy of DC combined with temporal pole resection in the treatment of patients with MCI, and to explore the ideal surgical treatment of patients with MCI, in order to provide some guidance and basis for the clinical treatment of patients with MCI.

## Materials and methods

### Clinical data

The clinical data of 158 patients with MCI from January 2015 to December 2018 were analyzed retrospectively. Follow the inclusion criteria and exclusion criteria, 41 patients were enrolled in the study. According to different surgical methods, the patients were divided into control group (DC) and study group (DC + temporal pole resection). All patients underwent unilateral DC. There were 18 cases in the control group, including 12 males and 6 females. The age ranged from 55 to 78 years, with an average age of 62.4 ± 4.4 years. There were 4 of left infarction and 14 cases of right infarction. There were 23 people in the study group, including 15 males and 8 females, aged from 52 to 75 years, with an average age of 62.3 ± 5.0 years. There were 6 cases of left infarction and 17 cases of right infarction. All patients were given dehydration and other medical treatment before operation, and the effect was poor. All methods were carried out in accordance with relevant guidelines and regulations.

### Inclusion criteria

1) Acute MCI confirmed by CT or MRI, and 2 / 3 of the blood supply area of the middle cerebral artery was involved. 2) Preoperative CTA confirmed the occlusion of large vessels in the supply infarct area. 3) The patient’s family members gave informed consent and signed on the operation method and risk.

### Exclusion criteria

1) Patients with previous history of stroke and residual serious sequelae; 2) Acute MCI of bilateral cerebral hemispheres; 3) CT showed that hemorrhage transformed after infarction and caused serious space occupying effect; 4) More than 72 hours after onset and mild clinical symptoms; 5) Bilateral mydriasis and severe impairment of brainstem function; 6) Severe complications and can’t tolerate the operation.

### The operation mode was as follows

DC is routinely performed in patients with MCI. If the preoperative CT shows that the midline deviation is greater than 1 cm, the annular cistern disappears, and the patient has malignant encephalocele during the operation, DC+ Temporal pole resection is performed.

#### Control group: DC

The patient was in supine position with his head leaning to the opposite side of the lesion. The skin incision starts from 1 cm in front of the tragus on the zygomatic arch, extends behind the auricle to 1 cm behind the parietal tubercle, and then extends to the hairline of the forehead along 1 cm next to the sagittal suture, in the shape of a question mark. The frontal, temporal and parietal bone flap with a diameter of about 14 cm was removed; The sphenoid ridge was flattened with bone biting forceps, the side was fully exposed, and the bone margin was flush with the middle cranial fossa; Suspended dura mater intermittently; The dura was cut radially, and when the brain tissue was no longer bulging out, the dura was tension-reducing suture, and the defect was repaired with artificial dura mater.

#### Study group

Temporal pole resection was performed on the basis of DC in the control group. Temporal pole resection was divided into dominant hemisphere and non dominant hemisphere. The temporal pole of the dominant hemisphere was excised into the anterior 4 cm temporal lobe, starting from the superior temporal gyrus, deep to part of the parahippocampal gyrus, and the bottom was within 4 cm from the temporal pole. Non dominant hemisphere decompression includes the anterior 6 cm temporal lobe, deep from the superior temporal gyrus to part of the parahippocampal gyrus, and the bottom is within 6 cm from the temporal pole. After complete hemostasis, the dura was tension-reducing suture, and the defect was repaired with artificial dura mater (Fig. 2).

All patients underwent ICP monitoring device implantation, and the ICP probe was implanted into the infarcted brain tissue in the operation area. After the operation, the vital signs were closely monitored, the head of the bed was raised by 30°, the airway was opened, and symptomatic support treatments such as analgesia, sedation, blood pressure management and prevention of infection were given. Different doses of osmotic therapy (mannitol) were given to reduce intracranial pressure according to the changes of intracranial pressure.

#### Observed indicators

Age, gender, underlying diseases, (such as hypertension, Heart disease, diabetes), admission GCS score, preoperative pupil, preoperative midline deviation, time from symptom to operation, NICU stay, changes of postoperative ICP were recorded. The patients were followed up for 6–12 months. The mortality rate and Modified Rankin scale (mRS) were recorded.

#### Statistical analysis

Data were analyzed using SPSS 22.0. Continuous variables were expressed as mean ± standard deviation or median. Differences between variables were evaluated using the independent samples t-test. Categorical variables were expressed as a ratio (%), and differences between variables were compared using the chi-squared test. Data were deemed significant if *P* < 0.05 for all tests.

## Result

There was no significant difference in age, previous diseases, systolic blood pressure, diastolic blood pressure, GCS and NIHSS scores between the two groups (Table 1).

**Table 1:** Comparison of general data between the two groups

	Control group (*n* = 18)	Study group (*n* = 23)	*P* value
Gender			0.213
Male	12	18	
Female	6	8	
Age (years)	62.4 ± 4.4	62.3 ± 5.0	0.537
Previous disease:			
Hypertension	13	18	0.239
Heart disease	5	7	0.263
Diabetes	3	2	0.639
GCS at admission	9.8 ± 1.1	9.3 ± 1.3	0.330
NIHSS at admission	18.5 ± 3.2	19.0 ± 1.3	0.723
Preoperative pupil			
No mydriasis	10	16	0.136
Unilateral mydriasis	8	7	0.087
Left hemisphere	4	6	0.579
Right hemisphere	14	17	0.160
Midline shift before DC			
<5 mm	3	5	0.807
5-10 mm	6	4	0.184
≥ 10 mm	2	3	0.907
Uncinate hernia before DC	9	12	0.839
Delay onset to DC(h)	36.4 ± 7.2	38.1 ± 6.9	0.173
Midline shift after DC			
<5 mm	10	18	0.177
5-10 mm	5	4	0.295
≥ 10 mm	3	1	0.070
NICU length of stay(d)	12.3 ± 1.6	9.2 ± 1.3	0.025
Hospital length of stay(d)	32.4 ± 2.1	25.2 ± 2.0	0.031

### NIHSS score

There was no significant difference in NIHSS score between the two groups before operation. Fourteen days after operation, the scores of the two groups were lower than those before operation, and the score of the study group was significantly lower than that of the control group, the difference was statistically significant (*P* < 0.05) (Table 2).

**Table 2:** Comparison of NIHSS scores before operation and 14 days after operation between the two groups

Groups	n	NIHSS score		*P* value
		Preoperative	Postoperative	
Control group	18	19.7 ± 2.3	9.5 ± 6.1	0.003
Study group	23	19.2 ± 3.3	6.5 ± 4.2	<0.001
*P* value		0.187	0.029	

### ICP monitoring

**Fig. 1:**
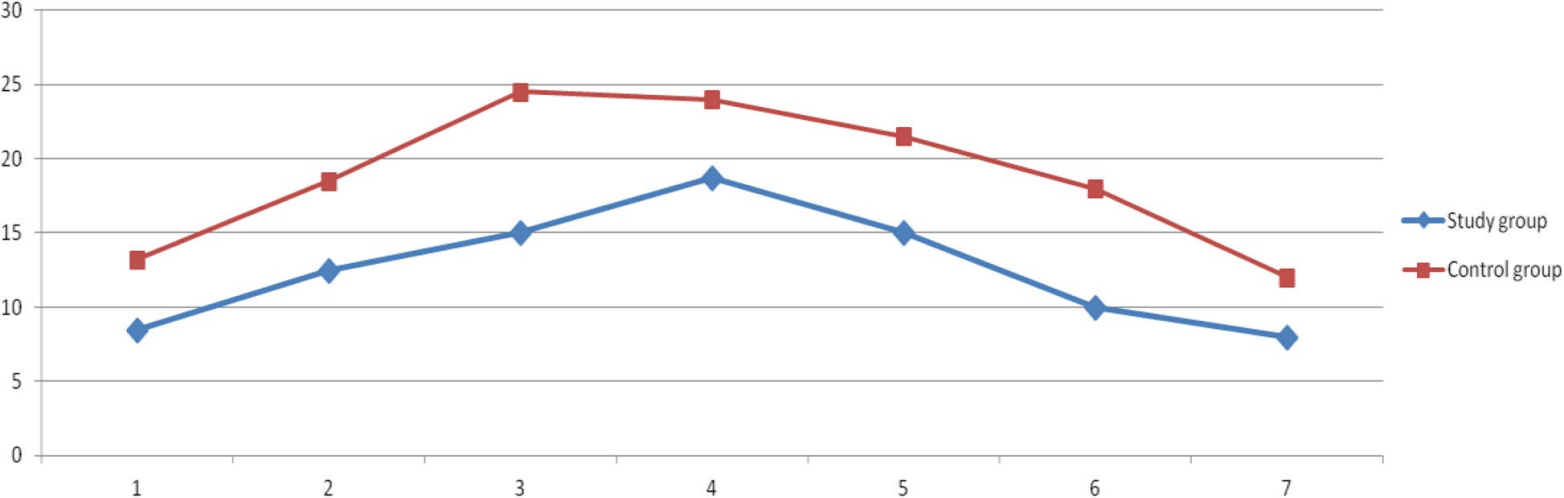
Comparison of ICP between the two groups within one week

After the operation, the patient returned to the NICU, the ICP monitoring device is externally connected to the monitor, it directly converts the ICP into digital and display on the display screen. Record the IC*P* value every half an hour and continuously monitored 7 days after operation. Exclude all factors causing ICP increase (such as: pain, fever, cough, urinary retention, irritability, etc.), if ICP ≥ 20mmhg, osmotic drugs shall be used to reduce intracranial pressure. The average value of ICP in each day after surgery was compared between the two groups. The ICP in the study group reached the peak on the fourth day after operation, and was treated with mannitol 125 ml, 12 h / time. The ICP in the control group reached the peak on the third day after operation, and was treated with mannitol 125 ml, 8 h / time. The ICP in both groups decreased on the fifth day after operation. During the monitoring of ICP, the ICP of the study group was significantly lower than that of the control group, and the dosage of osmotic therapy was less than that of the control group. The difference between the two groups was statistically significant (Fig. 1).

**Fig. 2:**
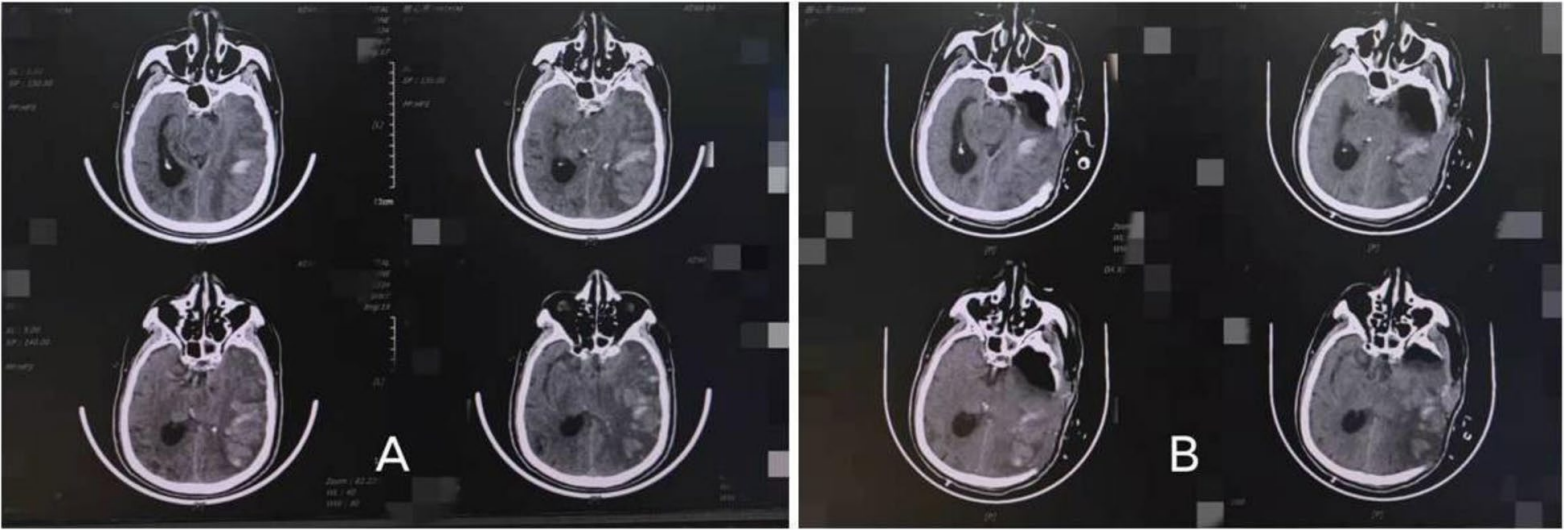
This patient underwent interventional thrombectomy for middle cerebral artery infarction and underwent DC + temporal pole resection after his condition deterioted. The high-density shadow in the infarct is contrast medium spillage. A: Preoperative, B: Postoperative

### Mortality

Five cases (27.8%) died in the control group one month after operation and 3 cases (13.0%) in the study group. There was significant difference between the two groups. During the follow-up, 2 cases died in the control group and 2 cases in the study group. After one-year follow-up, the total mortality of the control group was 38.8%, and that of the study group was 21.7%. The mortality of the study group was significantly lower than that of the control group. The difference between the two groups was statistically significant (Table 3).

**Table 3:** Comparison of mortality between the two groups

Times	Death	
	Control group(n;%)	Study group(n;%)
1 month after operation	5 (27.8)	3 (13.0)
3 months after operation	1 (5.5)	2 (8.7)
Half a year after operation	1 (5.5)	0 (0)
One year after operation	0 (0)	0 (0)
Total mortality	7 (38.8)	5 (21.7)

### mRS score

Among all the surviving patients, mRS score was performed 6 months after operation. If mRS<4, the prognosis is good, and if mRS ≥4, the prognosis is poor, and the good prognosis rate of the study group and the control group was more than 50%. The poor prognosis rate of the study group was significantly lower than that of the control group, but the difference between the two groups was statistically significant (Table 4).

**Table 4:** Comparison of mRS scores at 6 months after operation between the two groups of surviving patients

Groups	n	mRS scores					
		0	1	2	3	4	5
Control group(%)	18	0	0	1 (5.6)	5 (27.8)	3 (16.7)	2 (11.1)
Study group(%)	23	0	0	3 (13.0)	9 (39.1)	5 (21.7)	1 (4.3)
*P* value		0.034				0.004	

### Length of stay

Compared with the total length of stay in NICU of the two groups, the length of stay in the study group was significantly lower than that in the control group, and the difference between the two groups was statistically significant (Table 1).

## Discussion

Cerebral infarction accounts for about 80% of all stroke, [7] and MCI is the type of cerebral infarction with the worst prognosis. In China, 10–20% of hospitalized patients with cerebral infarction are severe MCI [8]. The treatment of MCI is difficult and the prognosis is poor. The mortality of conservative treatment is 60–80% [1], so the MCI is the main type of death of cerebral infarction [7]. The common cause of death in patients with MCI is intractable intracranial hypertension caused by malignant brain edema. Intracranial hypertension will form a vicious circle under the restriction of skull structure, resulting in extensive ischemia and hypoxia of brain tissue, and brain hernia leading to brain stem failure and death. There is no better method for conservative treatment except high-dose application of osmotic therapy. At this time, if the DC can be carried out in time to prevent the progressive increase of intracranial pressure, the prognosis of patients can be effectively improved [9]. DESTINY、DECIMAL and HAMLET showed that in patients with malignant middle cerebral artery infarction, DC within 48 hours of onset can significantly improve the 1-year survival rate [10–12].

Traditional DC is only to remove the frontal, temporal and parietal bone flaps with a diameter of about 12-14 cm, and cut the dura mater to make the swollen brain tissue bulge out, so as to avoid brain stem compression and obstruction of cerebrospinal fluid circulation. However, the space provided by removing the bone flap during the operation is often filled with swollen brain tissue. The course of brain tissue swelling will not be stopped due to the end of the operation. Therefore, after DC, some patients still have increased intracranial pressure due to the continuous aggravation of brain edema, malignant high intracranial pressure not only aggravates patients’ clinical symptoms and affects patients’ prognosis, but also leads to patients death in severe cases [13, 14].

Fulvio Tartara [15] treated MCI by improving the surgical method. The method is to take the temporal arc incision, small bone window craniotomy, and remove the infarcted temporal lobe (temporal pole) until the edge to open the basic cisterns (ambiens, carotic cisterns) to perform an extended CSF drainage the obtain the maximum possible brain relaxation, the bone flap was returned and fixed. The result was that the postoperative survival rate of MCI was 100%. Only one patient died of pulmonary embolism. Among all surviving patients, mRS ≤ 3 accounted for 53.3%. Although the results of study are very satisfactory, it may be related to the selected cases. Through clinical practice, we think that the purpose of surgical treatment of MCI is to reduce intracranial pressure, increase brain perfusion and save the brain tissue in the penumbra. The decompression effect of the author’s surgical method is limited, but the harm caused by frontal, temporal and parietal lobe brain swelling is ignored, its risk is also very high. We think that this surgical method is not suitable for most patients with MCI.

Therefore, on the basis of traditional DC combined with Fulvio tartara’s partial operation (temporal pole resection), it provides sufficient and maximum compensation space for the smooth and excessive postoperative brain swelling, so as to save the life of patients and obtain satisfactory curative effect. NIHSS score, postoperative intracranial pressure, mortality, total hospital stay and NICU hospital stay in the study group were significantly lower than those in the control group, and the difference was statistically significant.

Studies have proved that there is a negative correlation between the ICP and the poor prognosis of patients with cerebral infarction [16]. Iddo paldor et al. [17] concluded that ICP monitoring after MCI has great guiding significance for treatment. They set an ICP treatment threshold of 20 mmHg in postoperative patients with stroke, even after DC. Sang beom Jeon et al. [18] analyzed the changes of ICP within 24 hours after DC, they concluded that early evaluation of ICP changes and effective intervention can improve the prognosis of patients. Silvia Hern á ndez dur á n et al. [19] concluded that a threshold of 10 mmHg within the first 72 positive hours was a reliable predictor of mortality in MCI, with an acceptable sensitivity of 70% and high specificity of 97%. However, at present, there are few studies on the changes of ICP within 1 week after DC. The peak of ICP is generally 72 hours after DC. If the ICP is effectively reduced during this period, the mortality rate of patients will be greatly decreased. In this regard, we also agree with Silvia Hern á ndez dur á n. However, Silvia Hern á ndez dur á n did not make a detailed analysis of the specific causes of intracranial hypertension.

Our study shows that only DC is performed and the dura is cut, the swollen brain tissue will immediately bulge and fill the whole bone window. Although the ICP is not increase significantly within 24 hours after DC, the ICP will increase significantly 48–72 hours after operation, and even progress to malignant intracranial hypertension, which will eventually lead to the death of the patients. The reasons for this situation are as follows: 1) With the continuous progress of brain edema, the space provided by DC can not meet the degree of brain edema. 2) Results of the combined action of surgical trauma (i.e. muscle and scalp injury, swelling) and cerebral infarction edema. The swelling of the head muscle and scalp partially offset the removal of the space provided by the DC, the offset of compensatory space and the continuous increase of brain edema, resulting in the malignant increase of intracranial pressure. The second reason is often the main reason why intracranial pressure can not be effectively relieved 48–72 hours or even 96 hours after operation. In view of this reason, we performed temporal pole resection on the basis of DC, which effectively reduced ICP and avoided the disadvantage of insufficient decompression space caused by local tissue swelling.

Early DC in patients with MCI can reduce the mortality from 71 to 22%, or even lower, and double the chance of a favorable functional outcome (mRS score ≤ 3) from 21 to 43% [20], Barbara casolla et al. [21] concluded that the mortality within 30 days after DC is 16.5%. Among the patients who survived after 30 days, 8 patients (4.5%) died during the follow-up period of 1 year, and the good rate of mRS was 55.9%. Funchal et al. [22] performed DC and implantation of intracranial pressure probe in patients with MCI. Their results showed that the mortality of patients during hospitalization was 14.3%, the mortality of 6 months was 50%, and the survival patients were followed up for 6 months, and the mRS ≤ 4 was 50%. Ralph Rahme et al. [23] summarized through research that the mortality after DC of MCI was 24%, the mRS ≤ 3 was 41%, and the mRS = 4 was 47%. And through the investigation of patients and their caregivers, despite high rates of physical disability and depression, the vast majority of patients are satisfied with life and do not regret having undergone surgery. This is also in line with the Chinese traditional concept of “as long as people are there, everything is there”.

Our study concluded that within 1 month after DC, the mortality of the study group was 13.0% and that of the control group was 27.8%. The difference between the two groups was statistically significant. During the follow-up, 2 cases died in the control group and 2 cases died in the study group. One year after DC, the total mortality of the control group was 38.8%, and that of the study group was 21.7%. The mortality of the study group was significantly lower than that of the control group. The difference between the two groups was statistically significant. Among the surviving patients, mRS score was performed 6 months after DC. The good prognosis rate of the study group and the control group was greater than 50%. The poor prognosis rate of the study group was significantly lower than that of the control group, the difference between the two groups was statistically significant.

### Study limitations

This study had certain limitations. First, it was a nonrandomized, retrospective study. Therefore there may have been selection bias for the surgical technique. Second, this study examined a relatively small patient cohort that may not be representative of the MCI population. Hence further randomized research based on a large population and appropriate follow-up durations are required to provide more information about MCI.

## Conclusion

Through research, we concluded that patients with MCI with obvious intraoperative brain tissue bulge and postoperative malignant intracranial pressure increase may have better therapeutic effect of DC combined with temporal pole resection. The decompression effect is satisfactory, the postoperative intracranial pressure is well controlled, and the mortality of patients is significantly reduced. It has good clinical application value.

## Data Availability

The datasets used or analysed during the current study are available from the corresponding author on reasonable request.

## Abbreviations

DC: Decompressive Craniectomy
MCI: Massive Cerebral Infarction
ICP: Intracranial Pressure
NIHSS: National Institutes of Health Stroke Scale
mRS: Modified Rankin Scale
NICU: Neurology Intensive Care Unit
MRI: Magnetic Resonance Imaging
CT: Computed Tomography
CTA: Computed Tomography Angiography
SPSS: Statistical Product and Service Solutions
